# A Dynamic DNA Color Image Encryption Method Based on SHA-512

**DOI:** 10.3390/e22101091

**Published:** 2020-09-28

**Authors:** Shihua Zhou, Pinyan He, Nikola Kasabov

**Affiliations:** 1 Key Laboratory of Advanced Design and Intelligent Computing, Ministry of Education, School of Software Engineering, Dalian University, Dalian 116622, China; zhangzhengchin@gmail.com; 2 Knowledge Engineering and Discovery Research Institute, Auckland University of Technology, Auckland 1010, New Zealand; nkasabov@aut.ac.nz; 3 Intelligent Systems Research Center, Ulster University, Londonderry BT52 1SA, UK

**Keywords:** color image encryption, DNA coding, two rounds of permutation–diffusion, SHA-512

## Abstract

This paper presents a dynamic deoxyribonucleic acid (DNA) image encryption based on Secure Hash Algorithm-512 (SHA-512), having the structure of two rounds of permutation–diffusion, by employing two chaotic systems, dynamic DNA coding, DNA sequencing operations, and conditional shifting. We employed the SHA-512 algorithm to generate a 512-bit hash value and later utilized this value with the natural DNA sequence to calculate the initial values for the chaotic systems and the eight intermittent parameters. We implemented a two-dimensional rectangular transform (2D-RT) on the permutation. We used four-wing chaotic systems and Lorentz systems to generate chaotic sequences and recombined three channel matrices and chaotic matrices with intermittent parameters. We calculated hamming distances of DNA matrices, updated the initial values of two chaotic systems, and generated the corresponding chaotic matrices to complete the diffusion operation. After diffusion, we decoded and decomposed the DNA matrices, and then scrambled and merged these matrices into an encrypted image. According to experiments, the encryption method in this paper not only was able to withstand statistical attacks, plaintext attacks, brute-force attacks, and a host of other attacks, but also could reduce the complexity of the algorithm because it adopted DNA sequencing operations that were different from traditional DNA sequencing operations.

## 1. Introduction

With the advent of the big data era, numerous digital images, carrying a large amount of information, are generated daily. Accordingly, the security issues of digital images have become increasingly critical. Traditional data encryption algorithms [1], such as RSA, Data Encryption Standard (DES), and Advanced Encryption Standard (AES), however, are not suitable for image encryption because of their large data capacity and the strong correlation between pixel points. Therefore, researchers have begun to look for new solutions for image encryption [1].

As a result of the special characteristics of DNA, excellent parallelism, and large information density, DNA coding [2,3] is popular in image encryption research. Hu et al. [4] proposed DNA-based image cryptography by implementing the DNA cycle operation in the diffusion process, thereby overcoming the limitations of the DNA complementary operation. Chai et al. [5] proposed an image encryption algorithm by employing DNA coding for the diffusion of pixel values. Meanwhile, Liu et al. [6] proposed a remote-sensing image encryption scheme by utilizing a two-dimensional (2D) logistic map to generate DNA masks; this was then used to generate the DNA matrix. Enayatifar et al. [7] proposed a robust multiple-image encryption with a DNA sequence operation implemented to diffuse the image. Belazi et al. [8] proposed an efficient medical image encryption scheme based on the combination of chaotic systems and DNA computation. Huo et al. [9] proposed a two-round image encryption algorithm utilizing DNA complementary rules. Furthermore, Revathy et al. [10] proposed an authenticated biomedical image transaction based on DNA. Wang et al. [11] used the DNA sequence operation to diffuse the image. Chen et al. [12] proposed a DNA-based image encryption algorithm based on the combination of self-adaptive permutation–diffusion. Liu et al. [13] proposed a color image encryption based on the dynamic DNA and 4-D memresistive hyper chaos. Aashiq et al. [14] presented an image encryption method based on chaotic attractors; on the frequency domain they used the integer wavelet transform to encrypt the image while on the spatial domain they used the DNA sequence. Ballesteros et al. [15] presented a novel method that deviated from traditional schemes, in which variable-length codes based on the Collatz conjecture were used to transform the content of the image into unintelligible audio. Moreover, Ouyang et al. [16] proposed a color image encryption method using the memristive hyperchaotic system and DNA encryption, and Zhu et al. [17] reported an image encryption algorithm based on a matrix of Kronecker products and DNA operations over finite fields. Zhu et al. [18] constructed a five-dimensional continuous hyperchaotic system, and proposed an image encryption scheme based on the hyperchaotic system; this system adopted a dynamic DNA coding mechanism and classical scrambling diffusion encryption structure.

However, some of these DNA-based image cryptography methods pose risks. First, for some DNA-based image encryption schemes, their parameters of the chaotic maps remain unchanged. Second, dynamic DNA coding with different rules is more secure than using only a single rule. Third, a simple confusion or diffusion process is not secure enough. Fourth, an image encryption scheme is not secure enough if the key streams are independent of the plain images.

A secure image encryption scheme should utilize a dynamic permutation and dynamic diffusion process. Moreover, dynamic DNA coding utilizing all rules is more secure than using only a single DNA coding rule. Furthermore, selecting an appropriate chaotic system is also necessary. In addition, the key streams should be dependent of the plain image so that it can resist plaintext attacks. To address these limitations, we proposed a new image encryption algorithm with the structure of two rounds of permutation–diffusion by employing Secure Hash Algorithm-512 (SHA-512), two chaotic systems, dynamic DNA coding, DNA sequencing operations, and conditional shifting.

## 2. Materials and Methods

### 2.1. Lorenz System

In 1963, Lorenz tried to explain the unpredictable behavior of the weather by setting up a system of differential equations. In this paper, the image encryption scheme utilizes this system [4]:

(1) $$\{\begin{array}{c} {c_{1}}^{˙}=\alpha \left(c_{2}-c_{1}\right)\\ {c_{2}}^{˙}=\gamma c_{1}-c_{3}z-c_{2}\\ {c_{3}}^{˙}=c_{1}c_{2}-\beta c_{3} \end{array}$$

where α, β, and γ are the system parameters. When α = 10, β = 8/3, and γ = 28, the system is chaotic.

### 2.2. Four-Wing System

A four-wing system is a four-dimensional hyperchaotic system. The four-wing hyperchaotic system is defined as follows [19]:

(2) $$\{\begin{array}{c} x^{˙}=ax+byz\\ y^{˙}=cy+dz\\ z^{˙}=exy+kz+mxw\\ w^{˙}=ny \end{array}$$

where *a*, *b*, *c*, *d*, *e*, *k*, *m*, and *n* are the system parameters. When *a* = 8, *b* = −1, *c* = −40, *d* = 1, *e* = 2, *k* = −14, *m* = 1, *n* = −2, its Lyapunov exponents are *LE*1 = 1.3938, *LE*2 = 0.5096, *LE*3 = 0, and *LE*4 = −47.8986. Because there are two positive Lyapunov exponents, the system has hyperchaotic characteristics.

### 2.3. DNA Coding and Decoding Rule

A DNA sequence consists of four nucleic acid bases, A (adenine), G (guanine), C (cytosine), and T (thymine), which satisfy the Watson–Crick structure [20]. The structure of a DNA sequence is a binary string; on each side of the string, every two nucleic acid bases are complementary, following the rules that A and T are complementary and G and C are complementary. Based on the Watson–Crick structure, only eight combinations can be used for DNA coding [20]. These are listed in Table 1.

### 2.4. DNA Complementary Rules

The DNA complementary rule operation is popular for diffusing a DNA matrix. To satisfy the Watson–Crick structure of the DNA sequence, the complementary rules are defined as follows [21]:

(3) $$\{\begin{array}{c} x\neq L\left(x\right)\neq L\left(L\left(x\right)\right)\neq L\left(L\left(L\left(x\right)\right)\right)\\ x=L\left(L\left(L\left(L\left(x\right)\right)\right)\right) \end{array}$$

In Equation (3), x represents a DNA nucleic acid base. There are six complementary rules [15]:

*Rule 1: (AT)(TC)(CG)(GA) Rule 2: (AT)(TG)(GC)(CA) Rule 3: (AC)(CT)(TG)(GA) Rule 4: (AC)(CG)(GT)(TA) Rule 5: (AG)(GT)(TC)(CA) Rule 6: (AG)(GC)(CT)(TA)*.

In this paper, the complementary rules are defined as follows:

*B* = *DNA_complementary_operation(A,times,rules)*, (4)

where *A* and *B* are the nucleic acid base before and after the DNA complementary operation, respectively, and times denotes a matrix which indicates how many times the complementary operation is implemented on a nucleic base in the matrix A, and rules denotes a matrix about which rule is chosen for the operation of the DNA complementary operation.

### 2.5. DNA Cycle Operation

Hu et al. [4] defined another method for DNA matrix diffusion. We use this method in this paper. The DNA cycle function is defined as follows:

New nucleic acid base = *L*(original nucleic acid base, *h*), (5)

Original nucleic acid base = *L*_1(new nucleic acid base, *h*), (6)

where *L* is the function of the DNA cycle operation, and *h* is how many times the DNA cycle operation is performed on the original nucleic acid base to get the new nucleic acid base.

**Figure 1:**
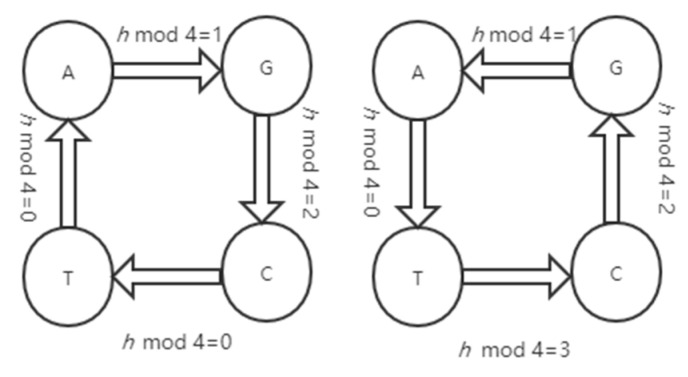
DNA cycle operation.

Figure 1 shows the process of DNA cycle operation and the inverse DNA cycle operation. To explain the Figure 1 in details, for instance, *L*(A, 3) = T, since mod(3, 4) = 3; and *L*_1(A, 7) = G, since mod(7, 4).

### 2.6. Mandelbrot Set

A Mandelbrot set is a plane in which all points belong to a complex plane and whose boundary forms a fractal. The Mandelbrot set is defined as **M**. Set **M** is used for the conditional shifting operation, which is defined later. A typical **M** set is defined as follows [22]:

(7) $$\underset{n\to \infty }{\lim}Z_{\left(n+1\right)}=Z_{n}^{2}+C$$

where $Z_{0}=0$.

In this paper, a modified Mandelbrot set is defined as follows:

(8) W(i,j)=(i×j)+C,

where W() denotes the Mandelbrot set **M**, i = 1,2, …, M and j = 1, 2, …, N, and the size of the image is M × N; C is constant, and C can be any large number. Considering the computational precision on Matlab, in this paper, set C = 10^14^, which is the most popular choice [22].

### 2.7. 2D-RT

To solve the limitation of the traditional Arnold maps (i.e., that it cannot permutate the non-square image), this paper used 2D-RT (two-dimensional rectangular transform). The improved 2D-RT can be defined as follows [23]:

(9) $$\left(\begin{array}{c} x^{\prime }\\ y^{\prime } \end{array}\right)=\left[\left(\begin{array}{cc} a & b\\ c & d \end{array}\right)\left(\begin{array}{c} x\\ y \end{array}\right)+\left(\begin{array}{c} r_{m}\\ r_{n} \end{array}\right)\right]mod\left(\begin{array}{c} m\\ n \end{array}\right)$$

and the inverse operation of the improved 2D-RT is expressed as

(10) $$\left(\begin{array}{c} x\\ y \end{array}\right)={\left(\begin{array}{cc} a & b\\ c & d \end{array}\right)}^{-1}\left(\begin{array}{c} x^{\prime }-r_{m}\\ y^{\prime }-r_{n} \end{array}\right)mod\left(\begin{array}{c} m\\ n \end{array}\right),$$

where *m* and *n* are the sizes of the image. Since 2D-RT was derived from the traditional Arnold map, 2D-RT was an enhanced Tent map and could permutate the non-square image. In this paper, the size of the RGB image *P* is transformed from *M* × *N* × 3 into *M* × 3N. 2D-RT is implemented *t* times to permutate the plain images. In Ref. [23], the system parameters *a*, *b*, *c*, and *d* satisfy *ad − bc* = 1. In the decryption process, we use the inverse matrix of the original matrix consisting of *a*, *b*, *c*, and *d*. In the encryption process:

(11) $$PST\left(x^{\prime },y^{\prime }\right)=P\left(x,y\right),$$

while in the decryption process:

(12) $$P\left(x,y\right)=PST\left(x^{\prime },y^{\prime }\right).$$

In this paper, *P* is the plaintext image. In the encryption process, the zero matrix *PST* with size *M* × 3*N* is defined in previous then the 2D-RT is performed on *P* for *t* times to generate the new matrix *PST* according to Equation (9).

## 3. Proposed Encryption Scheme

### 3.1. Initial Values and Intermittent Parameters

In the proposed scheme, SHA-512 is exploited and all the initial values of the chaotic system and the intermittent parameters are generated by the SHA-512 hash function of the plain image.

When the plain image is input, the hash sequence of the plain image with 512 bits is generated: K = [*k*1, *k*2, …, *k*64]. Next, the initial values are generated for the chaotic system.

First, *h*1, *h*2, *h*3, *h*4, *h*5, *h*6, and *h*7 are computed as follows:

(13) $$\{\begin{array}{c} h1=\frac{k1+k2+\cdots +k8}{8*256}\\ h2=1+\frac{k9⊕\ldots ⊕k16}{256}\\ h3=\frac{\left(k17⊕k18⊕k19⊕k20\right)+\left(k21⊕k22⊕k23⊕k24\right)}{2*256}\\ h4=\frac{\left(k25⊕k26⊕\ldots ⊕k32\right)}{256}\\ h5=\frac{\left(k33+k34+k35+k36\right)+\left(k37⊕k38⊕k39⊕k40\right)}{5*256}\\ h6=\frac{k41+k42+\cdots +k48}{8*256}\\ h7=\frac{\left(k49⊕k50⊕\ldots ⊕k56\right)+\left(k57+k58+\cdots k64\right)}{9*256} \end{array}$$

Second, one natural DNA sequence is selected, and then it is converted to a decimal number $d_{s}$. According to given values of four bases, the corresponding decimals of all bases in the DNA sequence are added. Then, the integer part of the product is removed, and the decimal part is retained. We can get the natural DNA sequence in http://www.ncbi.nlm.nih.gov/ according to geneID, the starting position and the length. For example, we chose a natural DNA sequence with the gene ID of 1054, the starting position of 1022, and the length of 17. The DNA sequence is {TGAAGTTTATACTGTAA}. Then, set A to 0.125112478141254, T to 0.58021545574585, C to 0.98754127451874, and G to 0.96148854586747. The corresponding decimals of all bases in the DNA sequence are added, and the sum is 8.68418997118962. Then, the integer part of 8.68418997118962 is removed, and the decimal part is retained. We can obtain $d_{s}=0.68418997118962$. Here, given values, the gene ID, the starting position and the length can all be regarded as part of the key, and they all are set manually.

Next, *h*1–*h*4 defined in Equation (13) and $d_{s}$ are used to calculate the initial values $x_{0}$, $y_{0}$, $z_{0}$, and $\omega_{0}$ for the hyperchaotic system, and are is defined as follows:

(14) $$\{\begin{array}{c} x_{0}=1+\frac{mod\left(\left(h1+h2+d_{s}\right)*10^{14},\;256\right)}{255}\\ y_{0}=1+\frac{mod\left(\left(h2+h3+d_{s}\right)*10^{14},\;256\right)}{255}\\ z_{0}=2+\frac{mod\left(\left(h3+h4+d_{s}\right)*10^{14},\;256\right)}{255}\\ w_{0}=2+\frac{mod\left(\left(h1+h2+h3+h4+d_{s}\right)*10^{14},\;256\right)}{255} \end{array}$$

Meanwhile, *h*5–*h*7 defined in Equation (13) and $d_{s}$ are used to calculate the initial values $c_{1}$, $c_{2}$, and $c_{3}$ for the Lorenz system:

(15) $$\{\begin{array}{c} c_{1}=1+\frac{mod\left(\left(h5+h6+d_{s}\right)*10^{14},\;256\right)}{255}\\ c_{2}=1+\frac{mod\left((h6+h7+d_{s})*10^{14},\;256\right)}{255}\\ c_{3}=2+\frac{mod\left(\left(h5+h6+h7+d_{s}\right)*10^{14},\;256\right)}{255} \end{array}$$

Finally, the intermittent parameters of $index_{1}$ to $index_{8}$ are calculated by the following:

(16) $$\{\begin{array}{c} index_{1}=mod\left(k33+k34+\cdots +k40,6\right)+1\\ index_{2}=mod\left(k41+k42+\cdots +k48,6\right)+1\\ index_{3}=mod\left(k49+k50+\cdots +k56,6\right)+1\\ index_{4}=mod\left(k57+k58+\cdots +k64,6\right)+1\\ index_{5}=mod\left(k33⊕k35⊕\ldots ⊕k63,6\right)+1\\ index_{6}=mod\left(k34⊕k36⊕\ldots ⊕k64,6\right)+1\\ index_{7}=mod\left(k1⊕k2⊕\ldots ⊕k64,6\right)+1\\ index_{8}=mod\left(k33⊕k34⊕\ldots ⊕k64,6\right)+1 \end{array}$$

According to Equations (13)–(16), all the initial values of the chaotic systems and the intermittent parameters were determined by the plain image. If there was a one-bit difference between two images, the initial values of the chaotic systems and the intermittent parameters were totally different. Moreover, the chaotic matrices and even the permutated plain images were totally different. Hence, the proposed scheme was sensitive to the plain image.

### 3.2. Conditional Shifting Operation

In this section, the Mandelbrot set is used for the conditional shifting operation. The conditional shifting operating is defined below Algorithm 1.
**Algorithm 1:** The Conditional Shifting Operation**Input**: Mandelbrot set **M** and the channels R_2_, G_2_, and B_2_.1:**for***I* = 1:*n*2:find the maximum value of *i*th column elements of **M** and denote it as *max_i_*3:find the maximum values of the *i*th row elements of R_2_, G_2_, and B_2_ and denote them as *max_ri_, max_gi_* and *max_bi_*, respectively, as follows:4:case 1:5:**if***max_i_* < *max_bi_*, **then**6:perform left cyclic shift on *i*th elements of R_2_ for max_i_ times7:else8:perform right cyclic shift on *i*th elements of R_2_ for max_i_ times9:**end if**10:**end**11:case 2:12:**if***max_i_* < *max_ri_* **then**13:perform left cyclic shift on *i*th elements of G_2_ for *max_i_* times14:**else**15:perform right cyclic shift on *i*th elements of G_2_ for *max_i_* times16:**end if**17:**end**18:case 3:19:**if***max_i_* < *max_gi_* **then**20:perform left cyclic shift on *i*th elements of B_2_ for *max_i_* times21:**else**22:perform right cyclic shift on *i*th elements of B_2_ for *max_i_* times23:**end if**24:**end**25:**end for**26:Finally, when the conditional shifting is finished, R_3_, G_3_, and B_3_ are obtained.

### 3.3. Whole Image Encryption Process

**Figure 2:**
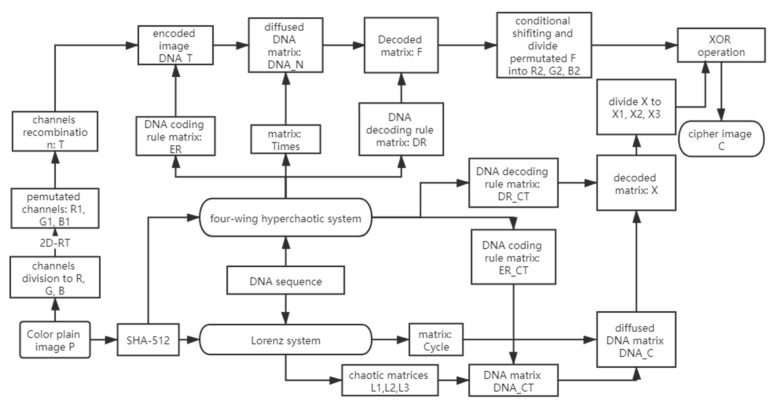
Flowchart of the proposed encryption scheme.

The complete encryption algorithm had a two-round permutation–diffusion structure. In the first round of the permutation–diffusion process, we implemented 2D-RT for permutating the plain image *P* for *t* times. Then we decomposed the permutated image *P* into R_1_, G_1_, and B_1_. The DNA complementary operation was used for the diffusion of the encoded plain image; meanwhile, the DNA cycle operation was implemented for the diffusion of the encoded chaotic matrices. In the second round of the permutation–diffusion process, the conditional shifting was implemented on the decoded images R_2_, G_2_, and B_2_, and we obtained the permutated matrices R_3_, G_3_, and B_3_. Finally, we used the decoded matrices XR, XG, and XB for diffusing matrices R_3_ and G_3_. The whole encryption process is demonstrated in Figure 2, and the encryption procedures are described in the subsequent subsections.

#### 3.3.1. First Round of Permutation

Step 1: Input the RGB plain image *P*_*M* × *N* × 3_.

Step 2: Make use of the plain image in the SHA-512 hash function to obtain the initial values for the chaotic systems and the intermittent parameters.

Step 3: Transform the plain image *P*_*M* × *N* × 3_ into *P*_*M* × 3*N*_. Perform 2D-RT on *P* to permutate the *Pt* times and obtain the PST.

Step 4: Divide the PST into three channels: R_1_, G_1_, and B_1_.

#### 3.3.2. Process of DNA Encoding

Step 1: Iterate the four-wing chaotic system, with the initial values of *x*_0_, *y*_0_, *z*_0_, and *w_0_*, 4*MN* + *l*0 times. Remove the first *l*0 terms to avoid the transient effect. Four sequences *X*, *Y*, *Z*, and *W* with the length of 4*MN* are obtained. Next, obtain the sequences *X*_1_, *Y*_1_, and *Z*_1_ by:

(17) $$\{\begin{array}{c} X_{1}=mod\left(\left(X+Y-fix\left(X+Y\right)\right)*10^{14},\;8\right)+1\\ Y_{1}=mod\left(\left(Y+Z-fix\left(Y+Z\right)\right)*10^{14},\;8\right)+1\\ Z_{1}=mod\left(\left(X+Y+Z-fix\left(X+Y+Z\right)\right)*10^{14},\;8\right)+1 \end{array}$$

*X*_1_ = [*x*_1_, *x*_2_, …, *x*_4MN_], *Y*_1_ = [*y*_1_, *y*_2_, …, *y*_4MN_], *Z*_1_ = [*z*_1_, *z*_2_, …, *z*_4MN_] are thus obtained.

Step 2: Iterate the Lorenz chaotic system, with the initial values of *c*_1_, *c*_2_, and *c*_3_, 4*MN* + l0 times. Remove the first *l*_0_ terms to avoid the transient effect. The three sequences *C*_1_, *C*_2_, and *C*_3_ with the length of 4*MN* are thus obtained. Next, we obtain the sequences *L*_1_, *L*_2_, and *L*_3_:

(18) $$\{\begin{array}{c} L_{1}=\mathrm{floor}\left(\mathrm{mod}\left(\left(\mathrm{abs}\left(C_{1}+C_{2}\right)-\mathrm{floor}\left(C_{1}+C_{2}\right)\right)*10^{14},\;256\right)\right)\\ L_{2}=\mathrm{floor}\left(\mathrm{mod}\left(\left(\mathrm{abs}\left(C_{1}+C_{3}\right)-\mathrm{floor}\left(C_{1}+C_{3}\right)\right)*10^{14},\;256\right)\right)\\ L_{3}=\mathrm{floor}\left(\mathrm{mod}\left(\left(\mathrm{abs}\left(C_{1}+C_{2}+C_{3}\right)-\mathrm{floor}\left(C_{1}+C_{2}+C_{3}\right)\right)*10^{14},\;256\right)\right) \end{array}$$

Step 3: Convert all the pixels of R_1_, G_1_, and B_1_ into binary numbers, and obtain three *M* × 8*N* matrices *R_bin*, *G_bin*, and *B_bin*. Then, recombine these three matrices into a single matrix *T* of 3*M* × 8*N* by *T* = *T_i_* (*i* = 1, 2, …, 6), where *i* = *index*_1_ and

$$T1\;=\;(\begin{array}{c} R\_bin\\ G\_bin\\ B\_bin \end{array});\;T2\;=\;(\begin{array}{c} R\_bin\\ B\_bin\\ G\_bin \end{array});\;T3\;=\;(\begin{array}{c} G_{\_}bin\\ R\_bin\\ B\_bin \end{array});$$

$$T4\;=\;(\begin{array}{c} G\_bin\\ B\_bin\\ R\_bin \end{array});\;T5\;=\;(\begin{array}{c} B\_bin\\ R\_bin\\ G\_bin \end{array});\;T6\;=\;(\begin{array}{c} B\_bin\\ G\_bin\\ R\_bin \end{array}).$$

Step 4: Transform the sequence *X*1, *X*2 and *X*3 into the *M* × 4*N* matrices and transform the sequence *L*1, *L*2 and *L*3 into the *M* × 4*N* matrices *L*_1, *L*_2 and *L*_3.

Step 5: Convert matrices *L*_1, *L*_2, and *L*_3 into binary matrices *L*1_*bin*, *L*2_*bin*, and *L*3_*bin* of *M* × 8*N*. Then recombine these matrices into a single 3*M* × 8*N* binary matrix *CT* by *CT* = *CTi*(*i* = 1, 2, …, 6), where *i* = *index*2 and

$$CT1=\;(\begin{array}{c} L1\_bin\\ L2\_bin\\ L3\_bin \end{array})\;CT2\;=\;(\begin{array}{c} L1\_bin\\ L3\_bin\\ L2\_bin \end{array})\;CT3\;=\;(\begin{array}{c} L2\_bin\\ L1\_bin\\ L3\_bin \end{array});$$

$$CT4\;=\;\left(\begin{array}{c} L2\_bin\\ L3\_bin\\ L1\_bin \end{array}\right)\;CT5\;=\;(\begin{array}{c} L3\_bin\\ L1\_bin\\ L2\_bin \end{array})\;CT6\;=\;(\begin{array}{c} L3\_bin\\ L2\_bin\\ L1\_bin \end{array}).$$

Step 6: The parameter index_3_ is used to construct two DNA encoding rule matrices *ER*_1_ and *ER*_2_ and *ER*_1_ = *ER*_*i*1_(*i*1 = 1, 2, …, 6), *ER*_2_ = *ER*_*i*2_(*i*2 = 1, 2, …, 6), *i*1 = *index*_3_, *i*2 = *mod*(*index*_3_, 6) + 1 and:

$${ER}_{1}\;=\;\left(\begin{array}{c} X\_1\\ Y\_1\\ Z\_1 \end{array}\right){ER}_{2}\;=\;\left(\begin{array}{c} X\_1\\ Z\_1\\ Y\_1 \end{array}\right){ER}_{3}\;=\;\left(\begin{array}{c} Y\_1\\ X\_1\\ Z\_1 \end{array}\right);$$

$${ER}_{4}\;=\;\left(\begin{array}{c} Y\_1\\ Z\_1\\ X\_1 \end{array}\right){ER}_{5}\;=\;\left(\begin{array}{c} Z\_1\\ X\_1\\ Y\_1 \end{array}\right){ER}_{6}\;=\;\left(\begin{array}{c} Z\_1\\ Y\_1\\ X\_1 \end{array}\right).$$

Step 7: For matrix *T*, recombine the eight binary planes by combing the first bit plane and the eighth bit plane into the bit plane matrix *T*18, and then do the same to the second bit plane and seventh bit plane, third bit plane and sixth bit plane, and fourth bit plane and fifth bit plane. Through this, we obtained the bit plane matrices *T*27, *T*36, and *T*45. The same operation is performed on the matrix *CT*, yielding matrices *CT*18, *CT*27, *CT*36, and *CT*45.

Step 8: The encoding rule matrix *ER*_1_ is used to encode matrices *T*18, *T*27, *T*36, and *T*45. *ER*_1_(1:2*M*,:) is used to encode matrix *T*18 and obtain DNA matrix *T_DNA*18. *ER*_1_(2*M* + 1:4*M*,:) is used to encode matrix *T*27 to obtain DNA matrix *T_DNA*27. *ER*_1_(4*M* + 1:6*M*,:) is utilized to encode matrix *T*36 to obtain DNA matrix *T_DNA*36. *ER*_1_(6*M* + 1:8*M*,:) is utilized to encode matrix *T*45 to obtain DNA matrix *T_DNA*45. Then the four DNA matrices are integrated into a single DNA matrix *DNA_T*: *DNA_T* = [*T_DNA*18, *T_DNA*27, *T_DNA*36, *T_DNA*45].

The encoding rule matrix *ER*_2_ is used to perform the same operation on matrices *CT*18, *CT*27, *CT*36, and *CT*45; and *ER*_1_ is used for matrices *T*18, *T*27, *T*36, and *T*45. Hence, DNA matrices *CT_DNA*18, *CT_DNA*27, *CT_DNA*36, *CT_DNA*45, and *CT_DNA* = [*CT_DNA*18, *CT_DNA*27, *CT_DNA*36, *CT_DNA*45] are obtained.

### 3.4. Diffusion and DNA Decoding

Step 1: Calculate the hamming distance *d*_1_–*d*_8_ by:

(19) $$\{\begin{array}{c} d_{1}=HD\left(T\_DNA18,T\_DNA27\right)\\ d_{2}=HD\left(T\_DNA27,T\_DNA36\right)\\ d_{3}=HD(T\_DNA36,T\_DNA45\\ d_{4}=\frac{d_{1}+d_{2}+d_{3}}{3}\\ d_{5}=HD\left(CT\_DNA18,CT\_DNA27\right)\\ d_{6}=HD\left(CT\_DNA27,CT\_DNA36\right)\\ d_{7}=HD\left(CT\_DNA36,CT\_DNA45\right)\\ d_{8}=\frac{d_{5}+d_{6}+d_{7}}{3} \end{array}$$

Update the initial parameters *x*_0_, *y*_0_, *z*_0_, *w*_0_, *c*_1_, *c*_2_, and *c*_3_ by:

(20) $$d_{1}^{\prime }=\frac{d_{1}}{3MN}\;d_{2}^{\prime }=\frac{d_{2}}{3MN}\;d_{3}^{\prime }=\frac{d_{3}}{3MN}\;d_{4}^{\prime }=\frac{d_{4}}{3MN},\;d_{5}^{\prime }=\frac{d_{5}}{3MN}\;d_{6}^{\prime }=\frac{d_{6}}{3MN}\;d_{7}^{\prime }=\frac{d_{7}}{3MN}\;d_{8}^{\prime }=\frac{d_{8}}{3MN},\;x_{0}^{\prime }=\frac{x_{0}+d_{1}^{\prime }}{2}\;y_{0}^{\prime }=\frac{y_{0}+d_{2}^{\prime }}{2}z_{0}^{\prime }=\frac{x_{0}+d_{3}^{\prime }}{2}\omega_{0}^{\prime }=\frac{z_{0}+d_{4}^{\prime }}{2},\;c_{1}^{\prime }=\frac{c_{1}+d_{1}^{\prime }+d_{2}^{\prime }}{2}\;c_{2}^{\prime }=\frac{c_{2}+d_{2}^{\prime }+d_{3}^{\prime }}{2}c_{3}^{\prime }=\frac{c_{3}+d_{3}^{\prime }+d_{4}^{\prime }}{2}.$$

Step 2: Utilize the updated initial parameters $x_{0}^{\prime },\;y_{0}^{\prime },\;z_{0}^{\prime },$ and $w_{0}^{\prime }$ to iterate the four-wing chaotic system 4*MN* + *l*1 times. Remove the first *l*1 times and obtain four sequences $X^{\prime }$, $Y^{\prime }$, $Z^{\prime }$, and $W^{\prime }$ of 4*MN*. Next, use $X^{\prime }$, $Y^{\prime }$, and $Z^{\prime }$ to generate sequences *X*_2_, *Y*_2_, and *Z*_2_:

(21) $$\{\begin{array}{c} X_{2}=mod(floor((X^{\prime }+Y^{\prime })-fix(X^{\prime }+Y^{\prime }))*10^{14},\;8)+1\\ Y_{2}=mod(floor((Y^{\prime }+Z^{\prime })-fix(Y^{\prime }+Z^{\prime }))*10^{14},\;8)+1\\ Z_{2}=mod(floor((X^{\prime }+Y^{\prime }+Z^{\prime })-fix(X^{\prime }+Y^{\prime }+Z^{\prime }))*10^{14},\;8)+1 \end{array}$$

Step 3: Convert sequences *X*_2_, *Y*_2_, and *Z*_2_ into matrices *X*_2, *Y*_2, and *Z*_2 of *M* × 4*N*. Next, use the intermittent parameter *index*_4_ and *mod* (*index*_4_, 6) +1 to construct and select the DNA decoding matrix by *DR_T* = *DR*_*i*1_, *DR_CT* = *DR*_*i*2_ (*i*1 = 1, 2, …, 6, *i*2 = 1, 2, …, 6), *i*1 = *index*_4_, *i*2 = *mod* (*index*_4_, 6) + 1 and:

$${DR}_{1}\;=\;\left(\begin{array}{c} X\_2\\ Y\_2\\ Z\_2 \end{array}\right)\;{DR}_{2}\;=\;\left(\begin{array}{c} X\_2\\ Z\_2\\ Y\_2 \end{array}\right)\;{DR}_{3}\;=\;\left(\begin{array}{c} Y\_2\\ X\_2\\ Z\_2 \end{array}\right)$$

$${DR}_{4}\;=\;\left(\begin{array}{c} Y\_2\\ Z\_2\\ X\_2 \end{array}\right)\;{DR}_{5}\;=\;\left(\begin{array}{c} Z\_2\\ X\_2\\ Y\_2 \end{array}\right)\;{DR}_{6}\;=\;\left(\begin{array}{c} Z\_2\\ Y\_2\\ Z\_2 \end{array}\right)$$

Step 4: Use the initial parameters $c_{1}^{\prime }$, $c_{2}^{\prime }$, and $c_{3}^{\prime }$ to iterate the Lorenz chaotic system 4*MN* + l1 times. Remove the first *l*1 terms to obtain the three sequences $C_{1}^{\prime }$, $C_{2}^{\prime },$ and $C_{3}^{\prime }$ of 4*MN*. $C_{1}^{\prime }$, $C_{2}^{\prime }$, and $C_{3}^{\prime }$ are used to obtain the three sequences $L_{1}^{\prime },\;L_{2}^{\prime }$, and $L_{3}^{\prime }$ by:

(22) $$\{\begin{array}{c} L_{1}^{\prime }=floor\left(mod\left(\left(abs\left(C_{1}^{\prime }+C_{2}^{\prime }\right)-floor\left(C_{1}^{\prime }+C_{2}^{\prime }\right)\right)*10^{14},\;256\right)\right)\\ L_{2}^{\prime }=floor\left(mod\left(\left(abs\left(C_{2}^{\prime }+C_{3}^{\prime }\right)-floor\left(C_{2}^{\prime }+C_{3}^{\prime }\right)\right)*10^{14},\;256\right)\right)\\ L_{3}^{\prime }=floor\left(mod\left(\left(abs\left(C_{1}^{\prime }+C_{2}^{\prime }+C_{3}^{\prime }\right)-floor\left(C_{1}^{\prime }+C_{2}^{\prime }+C_{3}^{\prime }\right)\right)*10^{14},\;256\right)\right) \end{array}$$

Step 5: Use $d_{5}^{\prime }$, $d_{6}^{\prime }$, $d_{7}^{\prime }$, and $d_{8}^{\prime }$ to update the initial parameters $x_{0}^{\prime },y_{0}^{\prime },\;z_{0}^{\prime }$, and $w_{0}^{\prime }$ to obtain $x_{0}^{\prime \prime },\;y_{0}^{\prime \prime },\;z_{0}^{\prime \prime }$, and $w_{0}^{\prime \prime }$ by:

(23) $$x_{0}^{\prime \prime }=\frac{x_{0}^{\prime }+d_{5}^{\prime }}{2}\;y_{0}^{\prime \prime }=\frac{y_{0}^{\prime }+d_{6}^{\prime }}{2}\;z_{0}^{\prime \prime }=\frac{z_{0}^{\prime }+d_{7}^{\prime }}{2}\;w_{0}^{\prime \prime }=\frac{w_{0}^{\prime }+d_{8}^{\prime }}{2}.$$

Then, use the updated initial parameters $x_{0}^{\prime \prime }$, $y_{0}^{\prime \prime },\;z_{0}^{\prime \prime }$, and $w_{0}^{\prime \prime }$ to iterate the four-wing hyperchaotic system 4*MN* + *l*2 times. Remove the first *l*2 terms and obtain the four sequences $X^{\prime \prime }$, $Y^{\prime \prime }$, $Z^{\prime \prime }$, and $W^{\prime \prime }$ of 4*MN*. Employ $X^{\prime \prime }$, $Y^{\prime \prime }$, and $Z^{\prime \prime }$ to calculate the new sequences $X_{3}$, $Y_{3}$, and $Z_{3}$:

(24) $$\{\begin{array}{c} X_{3}=mod(floor((X^{\prime \prime }+Y^{\prime \prime })-fix(X^{\prime \prime }+Y^{\prime \prime }))*10^{14},\;8)+1\\ Y_{3}=mod(floor((Y^{\prime \prime }+Z^{\prime \prime })-fix(Y^{\prime \prime }+Z^{\prime \prime }))*10^{14},\;8)+1\\ Z_{3}=mod(floor((X^{\prime \prime }+Y^{\prime \prime }+Z^{\prime \prime })-fix(X^{\prime \prime }+Y^{\prime \prime }+Z^{\prime \prime }))*10^{14},\;8)+1 \end{array}$$

Step 6: Transform sequences W, $W^{\prime }$, and $W^{\prime \prime }$ into *M* × 4*N* matrices W_1, W_2, and W_3, respectively. Then, use the intermittent parameter *index*_5_ to construct matrix *Times*, which is used in the DNA complementary operation to determine how many times the operation is performed on a nucleic acid base. *Times = Times_i_* (*i* = 1, 2, …, 6), *i = index*_5_ and:

$$Times_{1}=\left(\begin{array}{c} \begin{array}{c} W\_1\\ W\_2 \end{array}\\ W\_3 \end{array}\right)\;Times_{2}=\left(\begin{array}{c} \begin{array}{c} W\_1\\ W\_3 \end{array}\\ W\_2 \end{array}\right)\;Times_{3}=\left(\begin{array}{c} \begin{array}{c} W\_2\\ W\_1 \end{array}\\ W\_3 \end{array}\right)$$

$$Times_{4}=\left(\begin{array}{c} \begin{array}{c} W\_2\\ W\_3 \end{array}\\ W\_1 \end{array}\right)\;Times_{5}=\left(\begin{array}{c} \begin{array}{c} W\_3\\ W\_1 \end{array}\\ W\_2 \end{array}\right)\;Times_{6}=\left(\begin{array}{c} \begin{array}{c} W\_3\\ W\_2 \end{array}\\ W\_1 \end{array}\right)$$

The final matrix *Times* is calculated by:

*Times* = *mod*(*floor*(*Times* − *fix*(*Times*)) × 10^14^, 4) + 1 (25)

Step 7: Convert sequences $X^{\prime \prime }$, $Y^{\prime \prime }$, and $Z^{\prime \prime }$ into *M* × 4*N* matrices *X*_3, *Y*_3, and *Z*_3, respectively. Then, use the intermittent parameter *index*_6_ to construct and select the complementary rule matrix *CR*, which is used to determine which rule is selected in the DNA complementary operation. *CR* = *CR_i_* (*i* = 1, 2, …, 6), *i* = *index*_6_ and:

$$CR_{1}=\left(\begin{array}{c} \begin{array}{c} X\_3\\ Y\_3 \end{array}\\ Z\_3 \end{array}\right)\;CR_{2}=\left(\begin{array}{c} \begin{array}{c} X\_3\\ Z\_3 \end{array}\\ Y\_3 \end{array}\right)\;CR_{3}=\left(\begin{array}{c} \begin{array}{c} Y\_3\\ X\_3 \end{array}\\ Z\_3 \end{array}\right)$$

$$CR_{4}=\left(\begin{array}{c} \begin{array}{c} Y\_3\\ Z\_3 \end{array}\\ X\_3 \end{array}\right)\;CR_{5}=\left(\begin{array}{c} \begin{array}{c} Z\_3\\ X\_3 \end{array}\\ Y\_3 \end{array}\right)\;CR_{6}=\left(\begin{array}{c} \begin{array}{c} Z\_3\\ Y\_2 \end{array}\\ X\_1 \end{array}\right)$$

Step 8: Convert sequences $L_{1}^{\prime }$, $L_{2}^{\prime }$, and $L_{3}^{\prime }$ into matrices $L\_1^{\prime }$, $L\_2^{\prime }$, and $L\_3^{\prime }$, respectively. Then utilize the intermittent parameter *index*_7_ for the construction of the matrix *Cycle*, which is used to determine how many times the DNA cycle operation is performed on a nucleic acid base. *Cycle* = *Cycle_i_* (*i* = 1, 2, …, 6), *i* = *index*_7_ and:

$$Cycle_{1}=\left(\begin{array}{c} \begin{array}{c} L\_1^{\prime }\\ L\_2^{\prime } \end{array}\\ L\_3^{\prime } \end{array}\right)\;Cycle_{2}=\left(\begin{array}{c} \begin{array}{c} L\_1^{\prime }\\ L\_3^{\prime } \end{array}\\ L\_2^{\prime } \end{array}\right)\;Cycle_{3}=\left(\begin{array}{c} \begin{array}{c} L\_2^{\prime }\\ L\_1^{\prime } \end{array}\\ L\_3^{\prime } \end{array}\right)$$

$$Cycle_{4}=\left(\begin{array}{c} \begin{array}{c} L\_2^{\prime }\\ L\_3^{\prime } \end{array}\\ L\_1^{\prime } \end{array}\right)\;Cycle_{5}=\left(\begin{array}{c} \begin{array}{c} L\_3^{\prime }\\ L\_1^{\prime } \end{array}\\ L\_2^{\prime } \end{array}\right)\;Cycle_{6}=\left(\begin{array}{c} \begin{array}{c} L\_3^{\prime }\\ L\_2^{\prime } \end{array}\\ L\_1^{\prime } \end{array}\right)$$

Step 9: Perform the DNA complementary operation on matrix *T_DNA* to generate matrix *DNA_N:*

*DNA_N(i,j) = DNA_complementary_operation(T_DNA(i,j), Times(i,j), CR(i,j))* (26)

where *i* = 1, 2, …, 3*M* and *j* = 1, 2, …, 4*N*.

Step 10: Perform the DNA cycle operation on matrix *CT_DNA* to generate matrix *DNA_C*:

*DNA_C(i,j) = DNA_Cylcle_operation(CT_DNA(i,j), Cycle(i,j)),* (27)

where *i* = 1, 2, …, 3*M* and *j* = 1, 2, …, 4*N*.

Step 11: Utilize the DNA decoding matrix *DR_T* to decode DNA matrix *DNA_N*, which is further converted into decimal matrix *F* of 3*M* × *N*. Meanwhile, utilize DNA decoding matrix *DR_CT* to decode matrix *DNA_C*, which is further converted into decimal matrix X of 3*M* × *N*.

### 3.5. Second Round of Permutation and Diffusion

Step 1: Use intermittent parameter index_8_ to decompose matrix *F* into R_2_, G_2_, and B_2_ of *M* × *N*. *F_*1 = *F*(1:*M*,:), *F_*2 = *F*(*M* + 1:2*M*,:), *F_*3 = *F*(2*M* + 1:3*M*,:), *i* = *index*_8_, *i* = (1, 2, …, 6):

$$F_{1}:\left(\begin{array}{c} R_{2}\\ \begin{array}{c} G_{2}\\ B_{2} \end{array} \end{array}\right)=\left(\begin{array}{c} F\_1\\ \begin{array}{c} F\_2\\ F\_3 \end{array} \end{array}\right)\;F_{2}:\left(\begin{array}{c} R_{2}\\ \begin{array}{c} G_{2}\\ B_{2} \end{array} \end{array}\right)=\left(\begin{array}{c} F\_1\\ \begin{array}{c} F\_3\\ F\_2 \end{array} \end{array}\right)\;F_{3}:\left(\begin{array}{c} R_{2}\\ \begin{array}{c} G_{2}\\ B_{2} \end{array} \end{array}\right)=\left(\begin{array}{c} F\_2\\ \begin{array}{c} F\_1\\ F\_3 \end{array} \end{array}\right).$$

$$F_{4}:\left(\begin{array}{c} R_{2}\\ \begin{array}{c} G_{2}\\ B_{2} \end{array} \end{array}\right)=\left(\begin{array}{c} F\_2\\ \begin{array}{c} F\_3\\ F\_1 \end{array} \end{array}\right)\;F_{5}:\left(\begin{array}{c} R_{2}\\ \begin{array}{c} G_{2}\\ B_{2} \end{array} \end{array}\right)=\left(\begin{array}{c} F\_3\\ \begin{array}{c} F\_1\\ F\_2 \end{array} \end{array}\right)\;F_{6}:\left(\begin{array}{c} R_{2}\\ \begin{array}{c} G_{2}\\ B_{2} \end{array} \end{array}\right)=\left(\begin{array}{c} F\_3\\ \begin{array}{c} F\_2\\ F\_1 \end{array} \end{array}\right).$$

Meanwhile, use intermittent parameter *mod* (*index*_8_, 6) + 1 to decompose matrix X into XR, XG, and XB. *X*_1 = *X*(1:*M*,:), *X*_2 = (*M* + 1:2*M*,:), *X*_3 = *X*(2*M* + 1:3*M*,:), *i* = *mod*(*index*_8_,6) + 1, *i* = (1, 2, …, 6):

$$X_{1}:\left(\begin{array}{c} X_{R}\\ \begin{array}{c} X_{G}\\ X_{B} \end{array} \end{array}\right)=\left(\begin{array}{c} X\_1\\ \begin{array}{c} X\_2\\ X\_3 \end{array} \end{array}\right){\;X}_{2}:\left(\begin{array}{c} X_{R}\\ \begin{array}{c} X_{G}\\ X_{B} \end{array} \end{array}\right)=\left(\begin{array}{c} X\_1\\ \begin{array}{c} X\_3\\ X\_2 \end{array} \end{array}\right){\;X}_{3}:\left(\begin{array}{c} X_{R}\\ \begin{array}{c} X_{G}\\ X_{B} \end{array} \end{array}\right)=\left(\begin{array}{c} X\_2\\ \begin{array}{c} X\_1\\ X\_3 \end{array} \end{array}\right)$$

$$X_{4}:\left(\begin{array}{c} X_{R}\\ \begin{array}{c} X_{G}\\ X_{B} \end{array} \end{array}\right)=\left(\begin{array}{c} X\_2\\ \begin{array}{c} X\_3\\ X\_1 \end{array} \end{array}\right){\;X}_{5}:\left(\begin{array}{c} X_{R}\\ \begin{array}{c} X_{G}\\ X_{B} \end{array} \end{array}\right)=\left(\begin{array}{c} X\_3\\ \begin{array}{c} X\_1\\ X\_2 \end{array} \end{array}\right){\;X}_{6}:\left(\begin{array}{c} X_{R}\\ \begin{array}{c} X_{G}\\ X_{B} \end{array} \end{array}\right)=\left(\begin{array}{c} X\_3\\ \begin{array}{c} X\_2\\ X\_1 \end{array} \end{array}\right).$$

Step 2: Calculate the Mandelbrot set **M** by utilizing the introduced method. Use set **M** for the conditional shifting performed on R_2_, G_2_, and B_2_. Finally, R_3_, G_3_ and B_3_ are obtained.

Step 3: Obtain cipher image *C* by:

(28) $$\{\begin{array}{c} C\left(:,:,1\right)=R_{3}⊕X_{R}\\ C\left(:,:,2\right)=G_{3}⊕X_{G}\\ C\left(:,:,3\right)=B_{3}⊕X_{B} \end{array}$$

The proposed cryptosystem was symmetric. We decrypted the encrypted image by applying the encryption in reverse order. Note that we implemented the reverse DNA cycle operation, reverse DNA complementary operation, and reverse 2D-RT instead of the DNA complementary operation, DNA cycle operation, and 2D-RT, respectively. To decrypt the cipher image, the secret keys calculated by the SHA-2 algorithm instead of the hash code calculated by the SHA-2 are transmitted to another user for the decryption of the cipher images.

## 4. Stimulation Results and Security Analysis

### 4.1. Stimulation Results

In this section, we conducted stimulation experiments on Windows 7, with 4.00 GB RAM and an i5-4440 CPU. We implemented the scheme in Matlab 2017a (MathWorks, Natick, USA). Images 256 × 256 in size were used for the encryption and decryption: Lena, Pepper, Baboon, an all-black image, and an all-white image. The three images of objects were in color.

**Figure 3:**
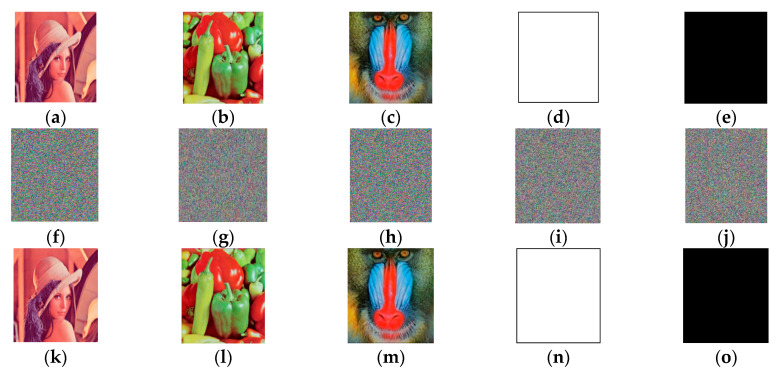
Stimulation results of the proposed scheme.

Figure 3a–e are plain images, Figure 3f–j are encrypted images, and Figure 3k–o are decrypted images. As demonstrated in Figure 3, the encrypted images were all noise-like images from which we could not obtain any useful information, but the decrypted images were identical to their plain images, which illustrated that the algorithm was secure and effective.

Additionally, Table 2 shows the system parameters of the 2D-RT, four-wing hyperchaotic system, and Lorenz chaotic system, and the abandoned numbers of the chaotic sequence. We selected one natural DNA sequence (GeneID is 154, and the starting position is 101, and the length is 1213.) to calculate the initial values. Aiming at the natural DNA sequence selected, we set A to 0.125112478141254, T to 0.58021545574585, C to 0.98754127451874, and G to 0.96148854586747. Figure 3a–e are the original images, and Figure 3f–j are the encrypted images corresponding to Figure 3a–e. Figure 3k–o are the decrypted images corresponding to Figure 3f–j.

### 4.2. Key Space Analysis

We used key space analysis to verify the image encryption scheme’s ability to resist brute-force attacks. According to [24,25], the key space must be larger than 2^100^ to guarantee the security of the image encryption scheme. In this paper, there are seven initial conditions. If the precision of the computer was 10^14^, the total key space was 10^14^^×^^7^ = (10^3^)^32.6^ ≈ (2^10^)^32.6^ = 2^326^. The key space of our algorithm is much larger than the theoretical value, so it can resist the exhaustive attack very well. Comparing our key space with others, our key space is also satisfactory. Table 3 shows the comparision of key space. From Table 3, it can be seen that our key space is as good as the key space of others’ algorithms, or even better.

### 4.3. Key Sensitivity Results

A secure encryption scheme is sensitive to a slight change of the keys. In the proposed encryption scheme, all the keys were generated from the SHA-512 hash function. Therefore, to test the key sensitivity of the proposed encryption scheme, we used the new hash value to change the last bit of the original hash value. The test image was Lena (256 × 256). In this paper, the hash value with the right key was denoted as K (K = 9f63791ec64b3bb5bcf1d6e1272557c9779b37575f33a72e0fbf7 3a8339bba94d0e3de2ab82ae305ee0a71a122123407227708ff0bc0296768566c2cc59e7d37), with the last bit changed being denoted as K1 (K1 = 9f63791ec64b3bb5bcf1d6e1272557c9779b37575f33a72e0fbf73a 8339bba94d0e3de2ab82ae305ee0a71a122123407227708ff0bc0296768566c2cc59e7d38) and the whole new hash value denoted as K2 (K2 = a1100bff91ac78cb8910aafcea1290fc99a3001cbbac73ef31ff23dd 1347f90 c60ad23fe26bd4133bad0501a273f0170adfe301261dc3df034ad00ff127526ff). The other encryption keys of the three experiments are the same. GeneID is 154, and the starting position is 22, and the length is 217. Set A to 0.98736273, T to 0.58021545, C to 0.1245737896434, and G to 0.0002356644.

**Figure 4:**
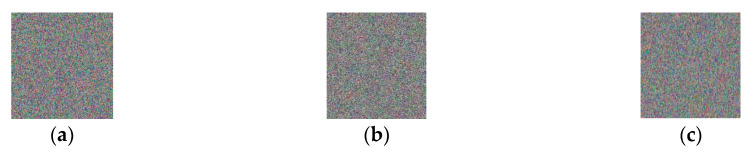
Key sensitivity results in the encryption process.

**Figure 5:**
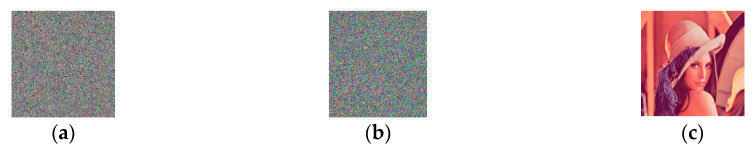
Key sensitivity results in the decryption process.

Figure 4a–c show the results obtained upon encrypting Figure 3a with K1, K2, and K, respectively. Figure 5a–c show the results when K was used to decrypt all encrypted images (Figure 4a–c), respectively. Table 4 lists *NPCR* (number of pixels change rate) values between the encrypted images with changed keys and the one with the right key. Table 5 lists *NPCR* values between the decrypted images with changed keys and the original image.

As the figures and tables show, a slight change in the original hash value or a whole new key leads to different encryption and decryption results. In Table 4 and Table 5, *NPCR* values with different keys are all close to the expected value of 0.9960, demonstrating that only the complete right hash value generates the right keys that can encrypt and decrypt the images correctly. Therefore, the proposed scheme is sensitive to a slight change in the hash value, which generates totally different keys and leads to totally different encryption and decryption results.

### 4.4. Correlation Analysis

Because of the strong correlation among pixels, the traditional encryption scheme could not be directly applied to the images [1]. However, a secure image encryption scheme could eliminate the correlation among pixels. In this section, we used the correlation coefficients to analyze the correlation among pixels between the plain image and encrypted image. Equations (29)–(31) are used to calculate the correlations between pixels in horizontal, vertical and diagonal directions.

**Figure 6:**
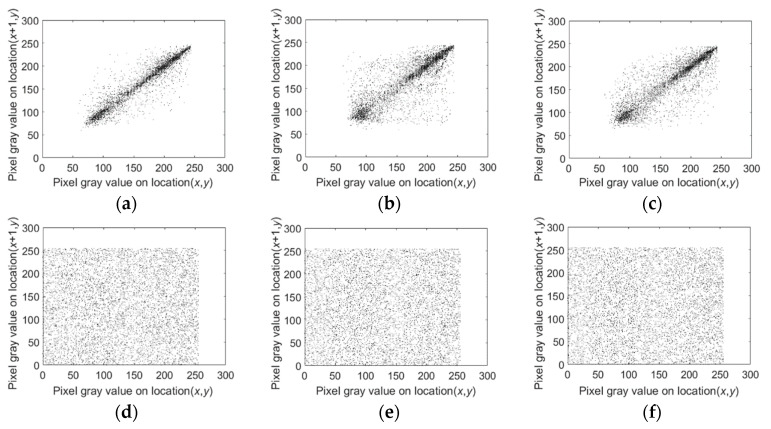
Correlation of two adjacent pixels in the R, G, and B channels for the plain and encrypted image Lena (256 × 256).

Figure 6 shows correlation of two adjacent pixels in the R, G, and B channels for the plain and encrypted image Lena in the horizontal direction. Figure 6a–c show correlations of the original image Lena in the R, G, and B channels respectively, and Figure 6d–f show correlations of the encrypted image Lena in the R, G, and B channels respectively. Figure 6 and Table 6 show that the correlation coefficients of the encrypted images are pretty low, and every pixel distributes evenly. From Table 7, we can see that the proposed scheme is comparable to other schemes in terms of correlation coefficients:

(29) $$r_{x,y}=\frac{E\left(\left(x-E\left(x\right)\right)\left(y-E\left(y\right)\right)\right)}{\sqrt{D\left(x\right)D\left(y\right)}},$$

(30) $$E\left(x\right)=\frac{1}{N}\sum_{i=1}^{N}x_{i},$$

(31) $$D\left(x\right)=\frac{1}{N}\sum_{i=1}^{N}{\left(x_{i}-E\left(x\right)\right)}^{2}.$$

### 4.5. Histogram Analysis

A secure image encryption scheme can resist statistical attacks. The elimination of correlation among pixels was necessary, and pixels of the encrypted image had to be distributed evenly. To verify whether the proposed scheme could distribute the encrypted image evenly, we conducted a histogram analysis.

**Figure 7:**
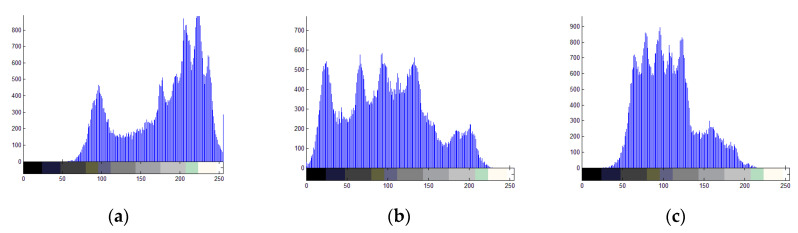

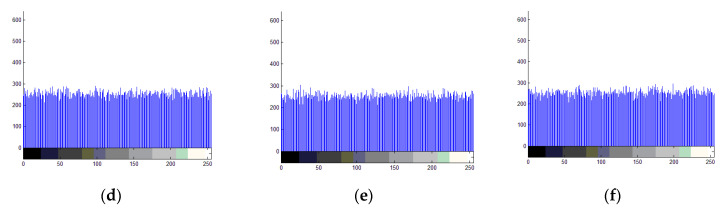
Histograms of the plain and encrypted image Lena.

Figure 7 shows the R, G, and B channels of the plain image Lena and its encrypted image with the size 256 × 256. Table 8 shows the variance of the constructed histograms (calculated by Equation (32)), and Table 9 compares our histograms with those produced by other schemes:

(32) $$var\left(X\right)=\;\frac{1}{n^{2}}\overset{N}{\underset{i=1}{\sum }}\overset{N}{\underset{j=1}{\sum }}\frac{1}{2}{\left(x_{i}-x_{j}\right)}^{2}$$

From Table 8, we can see that the variances of the original images are very high, while the variances of the encrypted images are greatly reduced. All encrypted variances are reduced by at least 98% compared to the original image variances. Figure 7 shows histograms of the plain and encrypted image Lena. Figure 7a–c are histograms of the plain image Lena in the R, G, and B channels, and Figure 7d–f are histograms of the encrypted image Lena in the R, G, and B channels, respectively, where x-axis denotes the pixel values in the image while y-axis denotes the frequency of the pixels in the image. From Figure 7, the histograms of the original images have obvious peaks, and histograms of the encrypted images are very uniform. Attackers cannot use a statistical attack to obtain any useful information by analyzing the histogram of the encrypted image. Therefore, our method can effectively resist statistical attacks. Table 9 shows comparison of histogram variance across methods about image Lena. It can be seen from Table 9 that our algorithm can obtain encrypted images with lower histogram variance.

### 4.6. Information Entropy Analysis

Information entropy is a metric that measures the randomness of an image and the amount of information hidden in an image:

(33) $$H\left(m\right)=-\overset{255}{\underset{i=0}{\sum }}P\left(x_{i}\right)\times log\;P\left(x_{i}\right).$$

Theoretically, a robust encryption scheme has an entropy value of 8. Table 10 shows the information entropy of the plain and encrypted images (size 256 × 256). The entropy is calculated by Equation (33). Our results were very close to 8, and thus were satisfactory. Table 11 compares information entropy across multiple schemes. Our algorithm was superior to other algorithms and was closer to the theoretical value of 8.

### 4.7. Differential Attacks and Chosen Plaintext Attack

Differential attacks crack the symmetric encryption scheme by analyzing the information distribution of the encrypted image. A secure symmetric encryption scheme is capable of resisting such attacks.

The *NPCR* and *UACI* (unified average change intensity) values of the R, G, and B channels are calculated as follows:

(34) $$NPCR=\frac{\sum_{i=1}^{N}\sum_{j=1}^{N}D\left(i,j\right)}{M\times N}\times 100\%,$$

(35) $$UACI=\frac{1}{255\times M\times N}\;\left[\overset{M}{\underset{i=1}{\sum }}\overset{N}{\underset{j=1}{\sum }}C\left(i,j\right)-C^{\prime }\left(i,j\right)\right]\times 100\%,$$

(36) $$D\left(i,j\right)=\{\begin{array}{c} 0,\;if\;C\left(i,j\right)=C^{\prime }\left(i,j\right)\\ 1,\;if\;C\left(i,j\right)\neq C^{\prime }\left(i,j\right) \end{array}.$$

Table 12 lists the *NPCR* and *UACI* values of encrypted images with a size of 256 × 256. Table 13 compares these values with those obtained through other schemes. As the tables illustrate, the *NPCR* and *UACI* of the R, G, and B channels were very close to the ideal values of 0.996 and 0.3346, respectively. Furthermore, the values of the proposed scheme were as good as the values obtained by the other methods. A secure and efficient encryption method is sensitive to a slight change in the plain image, and hence the encryption scheme is capable of resisting plaintext attacks. Usually, hackers employ all-black and all-white images to perform the chosen plaintext attack. As seen in Table 12, the NPCR and UACI of all-black and all-white images were close to the ideal values, thereby illustrating that the proposed scheme was sensitive to the plaintext and therefore could resist these attacks.

### 4.8. Noise and Occlusion Attack Analysis

During image transmission, noise and occlusion attacks are inevitable, but a robust encryption scheme can resist them. To verify whether the proposed scheme was capable of resisting noise and occlusion attacks, we used the encrypted image of Lena (256 × 256) as the test image. Salt-and-pepper noise (SPN) and Gaussian noise (GN) of varying intensities were added to the test image. In occlusion attack analysis, we added an occlusion effect to the test images; the occluding object occupied different proportions of the image and occurred at different positions.

In addition, we employed the peak-signal-to-noise ratio (*PSNR*) to calculate the difference between the original image and the decrypted images. The *PSNR* is calculated as follows:

(37) $$PSNR=10\times \log_{10}(\frac{255\times 255}{MSE})\;\left(dB\right),$$

(38) $$MSE=\frac{1}{MN}\;\overset{M}{\underset{i=1}{\sum }}\overset{N}{\underset{j=1}{\sum }}{\left[P_{1}\left(i,j\right)-P_{2}\left(i,j\right)\right]}^{2},$$

where *M* and *N* are the width and height of an image, respectively, and *P*1 and *P*2 are the original plain image and the image decrypted from the contaminated cipher image, respectively.

**Figure 8:**
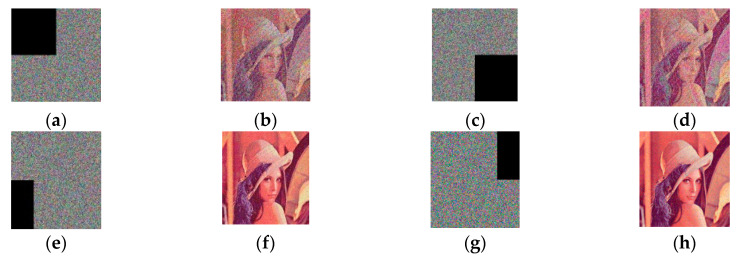
Stimulation results of occlusion attacks with Lena (256 × 256).

Figure 8 shows stimulation results of occlusion attacks with Lena. Figure 8a,c,e,g respectively represent the images obtained after Figure 3f suffered the different occlusion attack. Figure 8b,d,f,h respectively represent the decrypted images of Figure 8a,c,e,g Evidently, according to Figure 8 and Table 14, all PSNR values were larger than 27, and the decrypted images were all recognizable despite the various sorts of contamination in the encrypted images. Therefore, the proposed method was capable of resisting noise attacks and occlusion attacks.

### 4.9. Resistance to Some Typical Attacks

A secure cryptosystem should be capable of resisting cipher-text only attack, chosen-ciphertext attack, known-plaintext attack and chosen-plaintext attack. Among them, the chosen-plaintext attack is the most powerful. And if a cryptosystem is capable of resisting the chosen-plaintext attack, this cryptosystem is capable of resisting three other types of attack and we can declare that this cryptosystem is secure enough. In this paper, the encryption algorithm consists of two rounds of permutation-diffusion. In which, DNA encoding, DNA diffusion operation, DNA decoding, chaos and other techniques are used. And in our algorithm, the SHA-512 algorithm and the natural DNA sequence are used to generate the initial values of two chaotic systems. And the different image leads to the different initial values for the chaotic systems. Evidently, our algorithm is dependent on the plain image directly. In addition, if the hackers use the specific images such as all white and all black images to perform chosen-plaintext attack on our algorithm, the stimulation results of the all-white image and all-black image show that these two images all noise-like ones. Therefore, we can conclude that the proposed algorithm is capable of resisting the above mentioned typical attacks.

### 4.10. Contrast Investigation

Contrast investigation [31,32] is usually to calculate the local intensity variance in the image. Contrast is luminescence or color difference, through which objects in the image can be distinguished, and because the observer can recognize different objects. A higher contrast value indicates that the image has significantly different gray levels, while a constant gray level is represented by a lower value. Its mathematical description is:

(39) $$C=\underset{i,j}{\sum }{\left|i-j\right|}^{2}\times p\left(i,j\right)$$

where p(i,j) indicates the number of gray-level co-occurrence matices (GLCM).

Table 15 shows contrast values of plain images and encrypted images in R, G, B channels. From Table 15, contrast values of encrypted images are higher than ones of plain images. According to Ref. [31,32], it can prove that our method is satisfactory in terms of comparative investigation.

### 4.11. Energy

Energy calculations [31,32] result in the addition of square elements in GLCM. When the entries of GLCM are almost equal, the value of energy is lower, and when the amplitude of some entries is higher, the value of energy is higher. For encrypted images, the energy must be low:

(40) $$E=\underset{i,j}{\sum }p{\left(i,j\right)}^{2}$$

where p(i,j) indicates GLCM.

Table 16 shows energy values of plain images and encrypted images in R, G, B channels. According to Ref. [31,32], Table 16 can illustrate that encrypted images have the lower energy, and our method is satisfactory in terms of energy.

## 5. Conclusions

In this paper, we proposed an image encryption method with two rounds of permutation and diffusion. First, we employed the SHA-512 algorithm and the natural DNA sequence to generate the initial values for the four-wing hyperchaotic system and the Lorenz chaotic system, and the intermittent parameters. Since the hash value was determined by the plain image, a slight change in the plain image led to a totally different hash value so that the initial values for the chaotic system and the intermittent parameters were totally different, thereby leading to a totally different encrypted image in the end. Therefore, the proposed method was a one-time key pad scheme and was capable of resisting plaintext attacks. Second, we performed 2D-RT on the plain image *t* times. This was the first round of permutation. Since 2D-RT was derived from the traditional Arnold map, 2D-RT was an enhanced Tent map and could permutate the non-square image. Third, we employed the initial values to generate the chaotic sequences and the chaotic matrices for the construction of the DNA encoding rule matrices. All the DNA encoding rules depended on the plain image. Fourth, we used the intermittent parameters to construct the DNA matrices. Furthermore, the DNA matrices were used to calculate the hamming distances to update the initial values and iterate the chaotic systems for the second time, which eliminated the risk of using the secret keys several times. Fifth, the new chaotic matrices were generated, and the intermittent parameters were used to construct the DNA decoding rule matrices, making all the DNA decoding rules determined by the plain image. All of the rules were used in the first round of diffusion. In contrast to the traditional diffusion operations implemented on DNA matrices, in the proposed scheme, two different DNA diffusion operations were implemented on the encoded plain images and the encoded chaotic matrices: the dynamic DNA complementary rule operation and the DNA cycle operation. Finally, the eighth intermittent parameters were used to decompose the encoded images and encoded chaotic matrices, and in the second round of permutation–diffusion, we performed conditional shifting on the decomposed images and implemented the XOR calculation with the decomposed chaotic matrices to get the final encrypted image. Stimulation results and security analysis illustrated that the proposed scheme was secure and capable of resisting various sorts of attacks, and produced satisfactory stimulation results on image encryption and image decryption.

## Figures and Tables

**Table 1:** DNA encoding rules.

Rule	1	2	3	4	5	6	7	8
00	A	A	T	T	G	G	C	C
01	C	G	G	C	A	T	A	T
10	G	C	C	G	T	A	T	A
11	T	T	A	A	C	C	G	G

**Table 2:** System parameters in the proposed scheme.

Item	Value
System parameters of the four-wing hyperchaotic system	*a*= 8, *b* = −1, *c* = −40, *d* = 1, *e* = 2, *m* = 1, *n* = −2, *n* = −14
System parameters of the Lorenz chaotic system	*A* = 10, *β* = 8/3, *γ* = 28
System parameters of the 2D-RT	*A* = 1, *b* = 3, *c* = 5, *d* = 16, *r_m_* = 4, *r_n_* = 7, *t* = 5
Abandoned numbers of the sequence	*l*0 = 1000, *l*1 = 1000, *l*2 = 1000

**Table 3:** Comparison of key space.

Algorithm	Ours	Ref. [26]	Ref. [27]	Ref. [28]
Key space	10^98^	2^299^	10^9^^8^	10^9^^4^

**Table 4:** NPCR values of the encrypted images.

Image	Changed Key	R	G	B
Figure 4a	K1	0.9963	0.9962	0.9960
Figure 4b	K2	0.9963	0.9957	0.9957
Figure 4c	K	0	0	0

**Table 5:** NPCR values of the decrypted images.

Image	Changed Key	R	G	B
Figure 5a	K1	0.9965	0.9964	0.9956
Figure 5b	K2	0.9964	0.9962	0.9960
Figure 5c	K	0	0	0

**Table 6:** Correlation coefficients of the plain and encrypted image with the size of 256 × 256.

Image	Direction	Plain Image			Encrypted Image		
		R	G	B	R	G	B
	H	0.968	0.949	0.932	0.014	0.011	0.009
Lena	V	0.943	0.896	0.887	0.011	0.020	0.022
	D	0.918	0.859	0.852	0.035	0.016	0.021
	H	0.939	0.955	0.925	0.005	−0.015	−0.006
Pepper	V	0.931	0.935	0.905	0.029	−0.010	0.011
	D	0.887	0.894	0.842	0.012	−0.014	0.025
	H	0.917	0.919	0.938	−0.013	0.012	−0.011
	H	0.950	0.895	0.938	−0.014	−0.010	−0.004
Baboon	V	0.944	0.876	0.919	−0.022	0.014	−0.007
	D	0.921	0.827	0.889	−0.010	−0.018	0.021
All	H	#N/A	#N/A	#N/A	−0.011	0.015	0.012
black	V	#N/A	#N/A	#N/A	−0.021	−0.016	−0.016
	D	#N/A	#N/A	#N/A	0.016	−0.002	0.005
All	H	#N/A	#N/A	#N/A	0.001	0.002	0.003
white	V	#N/A	#N/A	#N/A	0.005	0.010	0.003
	D	#N/A	#N/A	#N/A	0.003	0.004	0.001

**Table 7:** Comparison of correlation coefficients across methods.

Algorithm	Encrypted Image			
	R	G	B	Average
Ours	0.0011	0.0018	0.0024	0.0018
Ref. [29]	−0.0027	0.0033	−0.0035	0.0031
Ref. [28]	0.0096	0.0109	0.0122	0.0109

**Table 8:** Histogram data of plain and encrypted images.

Image		Lena	Pepper	Baboon	All Black	All White
Plain image	R	76004.8672	57105.9766	22617.9609	#N/A	#N/A
	G	31563.3516	52138.7656	36848.7813	#N/A	#N/A
	B	95871.8906	103145.2813	35444.8828	#N/A	#N/A
Encrypted image	R	229.5391	259.8532	272.1654	263.6427	238.7628
	G	231.0976	249.9874	276.7468	263.9653	241.7543
	B	247.1986	264.4899	286.8965	255.3785	271.9436

**Table 9:** Comparison of histogram variance across methods about image Lena.

Algorithm	Variance		
	R	G	B
Ours	229.5391	241.9375	248.1328
Ref. [22]	249.7265	257.4453	256.1875
Ref. [29]	247.7800	279.6200	265.7100

**Table 10:** Information entropy of plain and encrypted images.

Image	Plain Image			Encrypted Image		
	R	G	B	R	G	B
Lena	7.1655	7.5578	6.8571	7.9974	7.9976	7.9975
Pepper	7.3009	7.5570	7.0929	7.9974	7.9973	7.9972
Baboon	7.6987	7.4251	7.5809	7.9970	7.9970	7.9971
All black	0.0000	0.0000	0.0000	7.9971	7.9971	7.9972
All white	0.0000	0.0000	0.0000	7.9974	7.9973	7.9970

**Table 11:** Comparison of information entropy across methods about image Lena.

Algorithm	Information Entropy		
	R	G	B
Ours	7.9974	7.9976	7.9975
Ref. [29]	7.9973	7.9969	7.9971
Ref. [27]	7.9973	7.9972	7.9969
Ref. [28]	7.9966	7.9972	7.9967

**Table 12:** *NPCR* and *UACI* values of different encrypted images.

Image	*NPCR*			*UACI*		
	R	G	B	R	G	B
Lena	0.9959	0.9960	0.9961	0.3354	0.3344	0.3345
Pepper	0.9962	0.9960	0.9959	0.3341	0.3339	0.3336
Baboon	0.9960	0.9961	0.9959	0.3345	0.3340	0.3334
All black	0.9961	0.9961	0.9958	0.3344	0.3345	0.3341
All white	0.9963	0.9959	0.9962	0.3344	0.3334	0.3351

**Table 13:** Comparison of *NPCR* and *UACI* values across methods about image Lena.

Image	*NPCR*			*UACI*		
	R	G	B	R	G	B
Ours	0.9959	0.9960	0.9961	0.3354	0.3344	0.3345
Ref. [29]	0.9960	0.9961	0.9961	0.3356	0.3345	0.3349
Ref. [28]	0.9961	0.9961	0.9961	0.3343	0.3343	0.3342
Ref. [30]	0.9963	0.9960	0.9960	0.3360	0.3330	0.3340

**Table 14:** *PSNR* results with Lena (256 × 256).

Item	R	G	B
GN with intensity = 0.02	28.2541	28.5421	28.3041
GN with intensity = 0.2	27.5014	27.3657	27.4251
SPN with intensity = 0.0002	57.4214	56.3527	56.8765
SPN with intensity = 0.0005	66.5047	67.4581	66.5041
SPN with intensity = 0.001	59.1021	61.1042	61.5384
1/8 data loss at the lower-left corner	30.6874	34.5478	35.6522
1/8 data loss at the upper-right corner	33.0001	33.0487	32.6894
1/4 data loss at the lower-right corner	31.5478	31.2587	31.3586
1/4 data loss at the upper-left corner	29.9564	32.7532	32.2287

**Table 15:** Contrast values of plain images and encrypted images in R, G, B channels.

Image	Plain Image			Encrypted Image		
	R	G	B	R	G	B
Lena	0.3672	0.3947	0.3405	10.5208	10.4763	10.5223
Pepper	0.1743	0.2341	0.1668	10.4999	10.4879	10.5112
Baboon	0.2248	0.2204	0.2430	10.5261	10.5012	10.4987

**Table 16:** Energy values of plain images and encrypted images in R, G, B channels.

Image	Plain Image			Encrypted Image		
	R	G	B	R	G	B
Lena	0.1391	0.0989	0.1756	0.0156	0.0156	0.0156
Pepper	0.1499	0.1183	0.1849	0.0156	0.0156	0.0156
Baboon	0.1047	0.1285	0.1233	0.0156	0.0156	0.0156

## References

[1] Huang H., He X., Xiang Y., Wen W., Zhang Y. (2018). A compression-diffusion-permutation strategy for securing image. Signal Process..

[2] Wang B., Zhang Q., Wei X. (2020). Tabu variable neighborhood search for designing DNA barcodes. IEEE Trans. NanoBiosci..

[3] Li X., Wang B., Lv H., Yin Q., Zhang Q., Wei X. (2020). Constraining DNA Sequences With a Triplet-Bases Unpaired. IEEE Trans. NanoBiosci..

[4] Hu T., Ouyang C.-J., Liu Y., Gong L.-H. (2016). An image encryption scheme combining chaos with cycle operation for DNA sequences. Nonlinear Dyn..

[5] Chai X., Chen Y., Broyde L. (2017). A novel chaos-based image encryption algorithm using DNA sequence operations. Opt. Lasers Eng..

[6] Liu H., Zhao B., Huang L. (2019). Aremote-sensing image encryption scheme using dna bases probability and two-dimensional logistic map. IEEE Access.

[7] Enayatifar R., Guimarães F.G., Siarry P. (2019). Index-based permutation-diffusion in multiple-image encryption using DNA sequence. Opt. Lasers Eng..

[8] Belazi A., Talha M., Kharbech S., Xiang W. (2019). Novel Medical Image Encryption Scheme Based on Chaos and DNA Encoding. IEEE Access.

[9] Huo D., Zhou D.-F., Yuan S., Yi S., Zhang L., Zhou X. (2019). Image encryption using exclusive-OR with DNA complementary rules and double random phase encoding. Phys. Lett. A.

[10] Revathy K., Thenmozhi K., Amirtharajan R., Praveenkumar P. (2018). CR Assisted IE Guarded Authenticated Biomedical Image Transactions. IEEE Photon. J..

[11] Wang X., Hou Y., Wang S.-B., Li R. (2018). A New Image Encryption Algorithm Based on CML and DNA Sequence. IEEE Access.

[12] Chen J.-X., Zhu Z.-L., Zhang L.-B., Zhang Y., Yang B.-Q. (2018). Exploiting self-adaptive permutation–diffusion and DNA random encoding for secure and efficient image encryption. Signal Process..

[13] Liu Z., Wu C., Wang J., Hu Y. (2019). A Color Image Encryption Using Dynamic DNA and 4-D Memristive Hyper-Chaos. IEEE Access.

[14] Banu S.A., Amirtharajan R. (2020). A robust medical image encryption in dual domain: Chaos-DNA-IWT combined approach. Med Biol. Eng..

[15] Ballesteros D.M., Peña J., Renza D. (2018). A Novel Image Encryption Scheme Based on Collatz Conjecture. Entropy.

[16] Ouyang X., Luo Y., Liu J., Cao L., Liu Y. (2020). A color image encryption method based on memristive hyperchaotic system and DNA encryption. Int. J. Mod. Phys. B.

[17] Zhu X., Liu H., Liang Y., Wu J. (2020). Image encryption based on Kronecker product over finite fields and DNA operation. Optik.

[18] Zhu S., Zhu C. (2020). Secure Image Encryption Algorithm Based on Hyperchaos and Dynamic DNA Coding. Entropy.

[19] Zhan K., Jiang W. (2017). Novel four-wing hyper-chaos system and its application in image encryption. Comput. Eng. Appl..

[20] Watson J.D., Crick F.H.C. (1953). Molecular Structure of Nucleic Acids: A Structure for Deoxyribose Nucleic Acid. Nature.

[21] Wang X., Wang Y., Zhu X., Luo C. (2020). A novel chaotic algorithm for image encryption utilizing one-time pad based on pixel level and DNA level. Opt. Lasers Eng..

[22] Jithin K., Sankar S. (2020). Colour image encryption algorithm combining Arnold map, DNA sequence operation, and a Mandelbrot set. J. Inf. Secur. Appl..

[23] Wu X., Zhu B., Hu Y., Ran Y. (2017). A novel color image encryption scheme using rectangular transform-enhanced chaotic tent maps. IEEE Access.

[24] Álvarez G., Li S. (2006). SOME BASIC CRYPTOGRAPHIC REQUIREMENTS FOR CHAOS-BASED CRYPTOSYSTEMS. Int. J. Bifurc. Chaos.

[25] Khan S., Han L., Lu H., Butt K.K., Bachira G., Khan N. (2019). A new hybrid image encryption algorithm based on 2D-CA, FSM-DNA rule generator, and FSBI. IEEE Access.

[26] Khan J.S., Boulila W., Ahmad J., Rubaiee S., Rehman A.U., AlRoobaea R., Buchanan W.J. (2020). DNA and Plaintext Dependent Chaotic Visual Selective Image Encryption. IEEE Access.

[27] Zhang Y., Xiao D. (2014). Self-adaptive permutation and combined global diffusion for chaotic color image encryption. AEU-Int. J. Electron. Commun..

[28] Rehman A.U., Liao X., Ashraf R., Ullah S., Wang H. (2018). A color image encryption technique using exclusive-OR with DNA complementary rules based on chaos theory and SHA-2. Optik.

[29] Chai X., Fu X., Gan Z., Lu Y., Chen Y. (2019). A color image cryptosystem based on dynamic DNA encryption and chaos. Signal Process..

[30] Wang X.-Y., Zhang H.-L., Bao X.-M. (2016). Color image encryption scheme using CML and DNA sequence operations. Biosystems.

[31] Qayyum A., Ahmad J., Boulila W., Rubaiee S., Masood F., Khan F., Buchanan W.J. (2020). Chaos-based Confusion and Diffusion of Image Pixels using Dynamic Substitution. IEEE Access.

[32] Masood F., Boulila W., Ahmad J., Arshad A., Sankar S., Rubaiee S., Buchanan W.J. (2020). A Novel Privacy Approach of Digital Aerial Images Based on Mersenne Twister Method with DNA Genetic Encoding and Chaos. Remote Sens..

